# Intravenous immunoglobulin: A potential treatment for the post-acute sequelae of SARS-Cov-2 infection?

**DOI:** 10.17305/bjbms.2021.6901

**Published:** 2022-02-12

**Authors:** Sandra E. Reznik, Amit K. Tiwari, Charles R. Ashby

**Affiliations:** 1 Department of Pharmaceutical Sciences, College of Pharmacy and Health Sciences, St. John’s University, Queens, New York, United States; 2 Departments of Pathology and Obstetrics and Gynecology and Women’s Health, Montefiore Medical Center/Albert Einstein College of Medicine, Bronx, NY, United States, United States; 3 Department of Pharmacology and Experimental Therapeutics, College of Pharmacy and Pharmaceutical Sciences, University of Toledo, Toledo, Ohio, United States; 4 Department of Cancer Biology, College of Medicine and Life Sciences, University of Toledo, Toledo, Ohio, United States

## POST-ACUTE SEQUELAE OF SARS-COV-2 (PASC)

Following the recovery from an initial infection with SARS-CoV-2, a recent meta-analysis of almost 1 million patients worldwide suggested that an estimated 43% of patients had experienced a variety of sequelae [1]. Furthermore, numerous studies indicate that patients can develop somatic, musculoskeletal, neuropsychiatric, cardiovascular, dermatologic, renal, hepatic, and gastrointestinal manifestations that are heterogeneous and vary in intensity and duration (for up to 12 months) [2,3]. The CDC has defined this phenomenon as “long-term symptoms that might be experienced weeks to months after primary infection with SARS-CoV-2, the virus that causes COVID-19 [4]” and proposed designations for this syndrome have included long COVID, long chronic COVID syndrome, chronic COVID, post-COVID-19 syndrome, post-acute covid-19 syndrome, and long-haulers [5]. On February 23, 2021, Dr. Francis Collins indicated that the persistent symptoms that occur in patients after recovery from the initial infection could be designated, PASC, which we will use in this article. At present, there is no clear or agreed on definition or diagnostic criteria for PASC. PASC is most likely a post-infectious illness similar to that reported by patients that had long-term sequelae that persisted for up to 14 months after infection with SARS-CoV-1 or the Middle Eastern respiratory SARS virus (MERS) [6]. Furthermore, post viral syndromes have been reported in patients who have recovered from the initial infection of non-coronaviruses, such as the Epstein-Barr virus, Ebola, dengue virus, west Nile virus, Zika virus, measles virus and poliovirus, among others [7]. In patients with PASC who tested negative for SARS-CoV-2, common symptoms include, but are not limited to, fatigue, myalgia, arthralgia, headache, dyspnea, cough, chest pain, alterations in taste and smell, diarrhea, anxiety, insomnia, depression, cognitive impairment, and dysautonomia [8,9]. Recent clinical studies indicate that PASC significantly affects the quality of life, exemplified by severe fatigue that decreases return to work and performance of routine daily tasks [10,11]. At present, the etiology of PASC remains to be elucidated. It has been hypothesized that PASC may be due to: (1) organ inflammation and damage that occurred in the acute infection stage; (2) a prolonged stay in an intensive care unit that can produce post-intensive care syndrome; (3) the exacerbation or unmasking of underlying comorbidities; (4) physical deconditioning and mental or psychosocial factors; (5) persistent, ongoing inflammation independent of the acute phase; (6) bystander activation and epitope spreading; (7) persistence of SARS-CoV-2 in cells or tissues; 8) reactivation of latent viruses and (9) inducing an autoimmune response due to molecular mimicry [12,13]. Interestingly, it has been hypothesized that PASC may result from the SARS-CoV-2 virus inducing autoimmunity, due to the formation of functional and diverse antibodies to SARS-CoV-2 that are cross-reactive with certain human antigens (i.e., autoantibodies), producing tissue inflammation and damage [14]. Indeed, data from clinical studies indicate that patients with PASC are significantly more likely to have circulating autoantibodies against a number of human antigens compared to healthy controls [15-17]. Furthermore, autoimmune diseases have been reported to occur in patients that had no previous history of autoimmune diseases after recovery from the initial SARS-CoV-2 infection, including but not limited to Guillain-Barre syndrome, Miller-Fisher syndrome, systemic lupus erythematosus, immune thrombocytopenia purpura, anti-phospholipid syndrome, Kawasaki’s disease, autoimmune hemolytic anemia, vasculitis, and multiple sclerosis [18]. Furthermore, certain viruses, for example, Epstein-Barr virus, human T-lymphocyte virus 1, hepatitis C, human parvovirus B19, Ebola, and human cytomegalovirus, have been reported to increase the risk of the development of autoimmune diseases [19].

## IVIG AS PROPOSED TREATMENT FOR PASC

At present, there are no efficacious treatments for PASC. As vaccines do not completely prevent the appearance of the PASC, there is still a need for alternative approaches to the treatment of the PASC. Clinical trials are ongoing to determine the efficacy of certain treatments for PASC; however, these trials remain to be completed. We hypothesize that patients diagnosed with PASC could be treated with intravenous immunoglobulin (IVIG). All IVIG products primarily consist of polyclonal IgG (predominantly monomeric IgG1 and IgG2 (at least 90% or greater) and lower levels of monomeric IgG3 (2–7%) and IgG4 (1–3%), with 1–10% dimeric complexes) [20]. The polyclonal IgG molecules are isolated from the blood of at least 3,000 up to 100,000 healthy blood donors [21]. These IgG molecules are produced by plasma B cells in response to immune stimuli [22]. Because IVIG is a human-derived blood product, there is the possibility of the transmission of pathogenic viruses and bacteria from the donors to the recipients. However, the risk of this is low as (1) microbial pathogens are removed using chemical and physical processes [23]; (2) the blood from all donors is screened for HIV-1, HIV-2, hepatitis B, hepatitis C, and syphilis [24]; and (3) neutralizing antibodies for several viruses are present in IVIG [25]. The pooled samples are representative of the environmental exposure of the donors to various pathogens and should contain antibodies that have multiple specificities for microbial antigens, self-antigens and anti-idiotypic antibodies [26]. IVIG is approved by the United States Food and Drug Administration (FDA) for the treatment of: (1) primary humoral immunodeficiency, bone marrow transplantation, pediatric HIV patients (to decrease the frequency of bacterial infections) infection and B-cell chronic lymphocytic leukemia and (2) chronic inflammatory demyelinating polyneuropathy, multifocal motor neuropathy, immune thrombocytopenic purpura and Kawasaki syndrome, which are believed to be autoimmune diseases [27]. In July 2021, the FDA, based on the results of ProDERM (ClinicalTrials.gov Identifier: NCT02728752) [28] approved the use of 10% Octagam for the treatment of dermatomyositis, a disease that has been hypothesized to result from an abnormal autoimmune response [29]. At present, in the U.S., there are thirteen approved IVIG products that are given intravenously, eight that are given subcutaneously and one that can be given intramuscularly [30]. Furthermore, randomized clinical trials indicate that IVIG has efficacy in certain neuromuscular, hematologic and dermatologic diseases that may result from alterations in autoimmunity and the inflammatory response [31].

## MECHANISM OF ACTION OF IVIG

The efficacy of IVIG, in certain autoimmune and inflammatory diseases, has been postulated to involve complex, mutually nonexclusive mechanisms. Broadly, the mechanism of action of IVIG involves the regulation of innate and acquired immunity by (1) the binding of the IgG antibodies to antigens via the Fab portion and (2) modulating the expression and function of immune cell Fc receptors [32]. Specifically, IVIG has been suggested to produce its therapeutic effect in certain autoimmune and inflammatory disorders as discussed below (for review of the mechanisms, see [26,32-35]). IVIG contains antibodies that interact with idiotypic determinants on pathogenic autoantibodies (anti-idiotype antibodies) that are associated with certain autoimmune diseases. The binding of the IVIG antibodies to the pathogenic autoantibodies inhibits their activation of (1) Fc receptor (FcγR) on immune cells and (2) complement, which decreases the activation of inflammatory pathways. The neonatal FcRs (FcRn), which are primarily located on endothelial cells, normally bind to IgG antibodies, which decreases their catabolism, increasing their half-life. At high doses, IVIG saturates FcRn, preventing the binding of endogenous pathogenic IgG antibodies to FcRns, thereby increasing their elimination. IVIG, by inhibiting the activation of macrophages and monocytes, can decrease inflammation by preventing the release of proinflammatory proteins (IL-1β, TNF-α) and increasing the levels of anti-inflammatory proteins (i.e., IL-10, TGF-β IL-1RA). Furthermore, IVIG has natural IgG antibodies that can interact with and neutralize the inflammatory effect of certain cytokines. IVIG alters the function and activity of dendritic cells (DC), which play a critical role in mediating autoimmune responses. IVIG decreases the activation of DC, inhibits the maturation and differentiation of human DC and decreases the processing and presentation of antigens by DC. IVIG can inhibit the proliferation and antigen-presentation functions of autoreactive B lymphocytes, thereby decreasing the levels of pathogenic autoantibodies present in patients with certain autoimmune diseases. IVIG can (1) neutralize B cell survival factors, such as BAFF and APRIL (increasing the likelihood of cell death), preventing the activation of FcγR; (2) decrease B cell proliferation; (3) sequester autoantigens and facilitate their clearance; (4) decrease B cell receptor-mediated activation of B-cells and (5) induce the apoptosis of autoreactive B cells, thus decreasing the levels of autoantibodies. IVIG, through the Fab2 portion of IgG, has been reported to inhibit T cell activation and proliferation. IVIG also induces the apoptosis of effector Th1 and Th17 cells by activating the Fas pathway. The expression of T regulatory cells and their suppressive effect on immune cells are increased by IVIG, thereby increasing immune tolerance. IVIG’s anti-inflammatory efficacy may also be due, in part, to its regulation of neutrophils. IVIG can decrease the recruitment and activation of neutrophils by inhibiting their interaction with the endothelium. *In vitro*, human neutrophil death can be induced by IVIG via its anti-sialic acid binding immunoglobulin-like lectins-9 (Siglec-9) autoantibodies. Certain proteins in IVIG can bind to the complement cascade molecules (1) C3b and C4b, preventing their interaction with the C5-C9 membrane attack complex; (2) C3a and C5a, and the anti-complement effect of IVIG could decrease the risk of inflammation and tissue damage.

## PROPOSED CLINICAL TRIAL

Based on the above information, we hypothesized that patients diagnosed with PASC could be treated with IVIG. IVIG’s efficacy could be evaluated in a random, double-blind placebo trial by identifying previously SARS-CoV-2-positive male and female patients (based on real time RT-PCR results) who are >18 years of age, whose symptom frequency and magnitude would be assessed before, during and after treatment, using the EuroQol 5-dimension, 3-level questionnaire [36]. A detailed medical history and tests for autoimmune diseases will be obtained for all patients to exclude other diagnoses. Pregnant women experiencing severe symptoms of PASC could be included in the trial, as IVIG is FDA pregnancy category C, generally considered safe in pregnancy in the medical community [37] and has been used to manage a variety of fetal and neonatal conditions [38]. Patients would receive either 2 g/kg of IVIG or an i.v. placebo, over 4–5 days, and be closely monitored in a health care setting. This high dose regimen should be used as it can regulate immune functions and produce significant anti-inflammatory efficacy [30]. Depending on the patients’ response, this regimen can be repeated every 4-6 weeks. Any patients that cannot tolerate the treatment due to any adverse effect will be removed from the trial. The infusion rate, depending on the concentration of the IVIG product, will be either 0.5 or 1 mg/kg/min or 0.5 mL/kg/hr for the first 10 minutes, and if there are no significant adverse or toxic effects, the infusion rate is increased to 0.5 mg/kg/min or 5 mL/kg/hr, respectively. The dosing interval will be determined by measuring the IgG trough level, a significant clinical response, or both. The primary efficacy point would be a significant decrease in the frequency and magnitude of the symptoms. IVIG formulations may not be comparable due to differences in pharmaceutical properties (i.e., pH, sugar content, sodium content, osmolality, and IgA content), which can affect their safety and tolerability [39], particularly in patients with certain morbidities.

## ADVERSE EFFECTS OF IVIG

The adverse effects produced by IVIG are rarely fatal. IVIG can produce flu-like symptoms, which include headache, abdominal pain, flushing, nausea, backache, fever, chills, myalgia and lethargy, which are typically mild, transient and occur within the first 30-60 minutes of administration [40]. Furthermore, decreasing the rate of infusion or temporarily stopping the infusion can alleviate these immediate adverse effects [41]. However, rare but serious adverse effects, such as thromboembolism, aseptic meningitis, thrombosis, arrhythmias, renal impairment, acute renal failure, delayed hemolysis, transfusion-related acute lung injury, antibody-dependent enhancement, and anaphylaxis, can occur [40]. The likelihood of adverse effects is increased (1) by a high concentration of IgA, Rh D antigen or anti-Rh blood group D [40]; (2) in patients that are IVIG naïve [42] and (3) have recently had a bacterial infection [42]. It has been postulated that the tolerability of IVIG during infusion may be proportional to the level of IgG dimeric aggregates and an increase in the levels of certain cytokines [43]. Clinical data suggest that the incidence and severity of these can be decreased by (1) assessing the patients’ health (detailed physical exam, medication use, blood work and review of past medical records prior to treatment; (2) infusing IVIG at a slow rate and (3) using appropriate premedications and prehydration [40]. The half-lives of IgG1, IgG2, IgG3 and IgG4 in IVIG in healthy adults are 23, 21, 21 and 7 days, respectively [44]. The serum half-life of IVIG products is 23-41 days [20]. High levels of IgG are present in the serum immediately after i.v. administration of IVIG, followed by a rapid decrease from 1 to 7 days [45]. IgG is primarily taken up into cells that express the neonatal Fc receptor (FcRn) and it is ultimately biodegraded in lysosomes to amino acids [46]. The immune response to live viral vaccines may be altered by IVIG products [24]. IVIG products are contraindicated in patients (1) that have previously had severe anaphylactic responses to IVIG and (2) with antibodies to IgA that have been diagnosed with IgA deficiency [20].

## CONCLUSION

In conclusion, we hypothesize that IVIG could attenuate the symptoms in patients diagnosed with PASC. Furthermore, therapies, such as aerobic exercise, psychological counseling, strength and balancing exercises, neuromuscular, respiratory, and cardiac interventions could also be used in patients with PASC. A multidisciplinary and multiprofessional team should be used to produce the maximal therapeutic outcome for each patient. Finally, if a patient cannot receive immune globulin by the intravenous route, the subcutaneous administration of immune globulins should be considered.
